# Haplotyping of *Cornus florida* and *C*. *kousa* chloroplasts: Insights into species-level differences and patterns of plastic DNA variation in cultivars

**DOI:** 10.1371/journal.pone.0205407

**Published:** 2018-10-23

**Authors:** Marcin Nowicki, Sarah L. Boggess, Arnold M. Saxton, Denita Hadziabdic, Qiu-Yun Jenny Xiang, Thomas Molnar, Matthew L. Huff, Margaret E. Staton, Yichen Zhao, Robert N. Trigiano

**Affiliations:** 1 Department of Entomology and Plant Pathology, The University of Tennessee, Knoxville, TN, United States of America; 2 Department of Animal Science, The University of Tennessee, Knoxville, TN, United States of America; 3 Department of Plant and Microbial Biology, North Carolina State University Raleigh, NC, United States of America; 4 Department of Plant Biology Rutgers, The State University of New Jersey, New Brunswick, NJ, United States of America; 5 Guizhou Key Laboratory of Agro-Bioengineering, Guizhou University, Huaxi, Guiyang, PRC; Austrian Federal Research Centre for Forests BFW, AUSTRIA

## Abstract

Chloroplast DNA is a part of plant non-nuclear genome, and is of particular interest for lineage studies. Moreover, the non-coding regions of cpDNA display higher mutation rates than the conserved coding cpDNA, which has been employed for phylogenetic and population research. We analyzed the cpDNA of 332 gDNA samples from collections of *Cornus florida* and *C*. *kousa* (commercial cultivars, breeding selections, and wild kousa accessions from Asia), using the chlorotyping system developed on North America-native, wild accessions of *C*. *florida*. Our results indicated significant differences in chlorotype frequencies between the two species. *Cornus florida* samples were represented by all major chlorotypes previously described, whereas all *C*. *kousa* samples analyzed had only one of the chlorotype patterns shown by *C*. *florida*. The chlorotyping analytic panel was then expanded by sequencing the targeted three non-coding cpDNA regions. Results indicated a major difference in the maternally-inherited cpDNA between the two closely related Big-Bracted *Cornus* species. Chlorotype diversity and differences in the proportion of informative sites in the cpDNA regions of focus emphasized the importance of proper loci choice for cpDNA-based comparative studies between the closely related dogwood species. Phylogenetic analyses of the retrieved sequences for the other species of *Cornus* provided information on the relative utility of the cpDNA regions studied and helped delineate the groups (Big-Bracted, Cornelian Cherries, Blue/White-Fruited) within the genus. Genealogical relationships based on the cpDNA sequences and the inferred chlorotype networks indicated the need for continued analyses across further non-coding cpDNA regions to improve the phylogenetic resolution of dogwoods.

## Introduction

Native to temperate and boreal climates of Europe, Asia, and North America, dogwoods (genus *Cornus* L.: Cornaceae) include up to 60 species of shrubs and small trees popular for ornamental and commercial uses (historically), wildlife and human food, and for some ethnic medicinal properties[1, 2]. Flowering dogwood, *C*. *florida* L., is native to the eastern United States of America (USA) and has been used as a model tree species in studies on plant biology[3, 4], biochemistry[5, 6], pathology[7, 8], and population genetics[9, 10]. Closely related *C*. *kousa* F. Buerger *ex* Hance F of Asian origin (China, Korea, and Japan) was naturalized to the USA mainly for ornamental purposes (including hybridization with *C*. *florida*) because of its attractive growth habit and showy bracts, and its overall high resistance to insects and pathogens[7, 8, 11–13]. The ornamental value of both species drove consumer demand and resulted in selection/breeding and commercial release of over 80 cultivars and 24 varieties for *C*. *florida*, and over 70 cultivars for *C*. *kousa*[14, 15], despite a relatively long breeding process[4, 16]. Both species are economically important in the USA with sales around $30M per year (corresponding to over 3 million trees sold). Tennessee-based growers accounted for about 25% of this value or almost one-half of the trees in commerce[17].

A population study[18] explored the chloroplast DNA (cpDNA) diversity in the wild accessions of *C*. *florida* across its native range utilizing three non-coding regions (*trnQ-rsp16*; *ndhF-rpl32*; *rsp16*). They found four different cpDNA haplotypes (chlorotypes; H), H1 to H4, with H1 being the most prevalent[18]. Their work also gave rise to an economical and convenient chlorotyping system utilizing PCR of three non-coding cpDNA regions and four restriction digests. This system saved time and resources on analyses of gDNA samples from the wild *C*. *florida* accessions providing substantial insights into the evolution, expansion, and current genetic structure of the species natural populations. Their diligent analyses focused on wild *C*. *florida* accessions using three non-coding cpDNA regions; many more such regions of potential interest were noted in literature[19, 20]. But, neither *C*. *florida* cultivars nor other related *Cornus* species were analyzed in their study30 nor elsewhere in this scope. Also, despite several cpDNA and nuclear regions used for *Cornus* genus phylogenetic studies[2, 21–23], relationships among a few species within the Big-Bracted and the Blue-/White-Fruited groups remain only partly resolved (reference [1] describes the clades within the *Cornus* genus and their etymology). To achieve this goal, one first needs to generate information regarding the relative utility of respective regions being tested (nuclear/cpDNA) to effectively resolve species relationships within these groups.

To fill this informational gap, the overarching goal of our project was to compare the chlorotype diversity (a proxy for cpDNA sequence variability) across three cpDNA regions of wild *C*. *florida* populations[18] with cultivars, breeding selections, and wild accessions of the Big-Bracted dogwood species, *C*. *florida* and *C*. *kousa*. It was expected that the cpDNA diversity in the wild *C*. *florida* accessions was greater than that in the cultivars of the same species. Specific objectives of our study were the following: (1) to contrast the chlorotype diversity of wild *C*. *florida* accessions[18] with this demonstrated by cultivars and breeding selections of *C*. *florida* and (2) to assess the utility of the three non-coding cpDNA regions for chlorotyping cultivars and wild accessions of *C*. *kousa*, a species closely related to *C*. *florida* from the Big-Bracted clade and for phylogenetic study to resolve the species relationships within the *Cornus* genus.

## Materials and methods

### Sampling and gDNA extraction

Samples of *C*. *florida* and *C*. *kousa* trees (cultivars, breeding selections, and trees of uncultivated, wild accessions) were obtained from different geographical locations across North America and Asia. No specific permissions were required for these locations/activities, as the majority of the materials were the cultivated *C*. *florida* and *C*. *kousa*. Neither of these two species is considered endangered and/or protected. Many of the samples originated from the collections held at the University of Tennessee, Knoxville, TN, USA (UT), and nurseries local to the region (Commercial Nursery, Decherd, TN, USA; Walnut Hill Nursery, Belvidere, TN, USA; Hidden Hollow Nursery, Belvidere, TN, USA). Plant material was also collected from Rutgers University, New Brunswick, NJ, USA. Additional samples of wild *C*. *kousa* accessions of direct Asian origin were provided to us by the Morton Arboretum, Lisle, IL, USA and the U.S. National Arboretum, Beltsville, MD, USA, upon our request. Isolated total DNA (gDNA) from wild Asian *C*. *kousa* accessions from North Carolina State University, Raleigh, NC, USA, and gDNA samples of other *Cornus* species from previous studies[24] stored at -80°C were also included.

In this study, we analyzed a total of 332 gDNA samples (see S1 Table). These samples included a group of 91 *C*. *florida* cultivars and breeding selections (44 cultivars, *i*.*e*., roughly half of those that are available commercially; a total of 132 *C*. *florida* samples), a group of 85 *C*. *kousa* cultivars or hybrids (63 cultivars, *i*.*e*., roughly 80% of commercially available), and 24 wild *C*. *kousa* accessions of Asian origin (China: *n* = 19; South Korea: *n* = 5) amounting to a total of 168 *C*. *kousa* samples. Our collection also included a *Cornus* spp. group of 17 other *Cornus* species[24] comprised of 32 samples with several species having multiple representations. The sampled cultivars/accessions partially overlapped among the origins, which constituted an internal control for the entire collection. The previously published data set of Call et al.[18], which included the chlorotyping results for 225 samples of wild *C*. *florida* accessions (here denoted as wild *C*. *florida* group), was also used in this study to compare with our chlorotyping data set of the *C*. *florida* group of cultivars and breeding selections.

gDNA was extracted from dormant flower buds or not fully expanded leaves using QIAGEN DNeasy Plant Mini kit protocol (Qiagen, Germantown, MD, USA), modified by the addition of 2% w/v Polyvinylpyrrolidone to the Lysis buffer, and an increased time of incubation at 4°C. Genomic DNA yield and purity were determined using a Nanodrop ND-1000 UV/Vis (Fisher Scientific, Pittsburgh, PA, USA) and gDNA quality was evaluated using 2% low melting point agarose gel electrophoresis and visualized with ethidium bromide.

### PCR amplification, plastid DNA restriction site analyses, and sequencing

The three following cpDNA non-coding regions were examined: *trnQ-rps16* (cpDNA01), *ndhF-rpl32* (cpDNA02), and *rps16* (cpDNA03) ([18, 20, 25]; S2 Table). Primers were synthesized by Integrated DNA Technologies (Coralville, IA, USA) and had been used previously for dogwood genetic diversity research30. Twenty μl PCR reactions for cpDNA01 and cpDNA03 used 0.5 μM of each primer and 10 ng of gDNA following the recommended protocol for AccuStart II PCR SuperMix (Quantabio, Beverly, MA, USA). The cpDNA02 proved more demanding with the same kit and required 1 μM of each primer and 20 ng gDNA for successful amplification (S2 Table). Restriction enzymes were purchased from Fisher Scientific and New England Biolabs (Ipswich, MA, USA). Products of the PCR reactions for all gDNA samples used for this study were electrophoresed on 2% agarose gels with ethidium bromide stain at 100 V/cm2 for 1 h. After ensuring amplification using this method, the PCR products were subjected to a series of restriction digests that were electrophoresed on 2% agarose gels with ethidium bromide stain at 100 V/cm2 for 1 h. Analytical digests were run for 1 h at the recommended temperatures for each of the various enzymes, in a volume of 15 μl, which included 10 μl of the PCR product, 1.5 μl of the respective enzyme’s 10 × buffer, 3 μl of 5 × loading dye, and 0.5 μl of the enzyme (approx. 5 U per reaction). Because cpDNA sequences are conserved, low variation in the amplicons of interest was expected. Point mutations in the regions provided access to restriction-driven analyses that can reveal differences between individuals as well as species. Chlorotypes for each gDNA sample were defined as binary data strings (0 for no digestion at given site or 1 for digested PCR product), which resulted from the restriction pattern using a series of enzymes.

To extend the chlorotyping panel (*i*.*e*., the number of informative nucleotide substitutions), the cpDNA01, 02, and 03 regions of six *C*. *kousa* cultivars, four *C*. *florida* cultivars, and one *C*. *florida* breeding selection (Table 1) were selected for sequencing. Collectively, these covered a broad range of chlorotyping variation in *C*. *florida* (no such variation was initially observed for *C*. *kousa*) and provenances within each species. The cpDNA01, 02, and 03 regions from samples of other *Cornus* species[24], belonging to three phylogenetic groups as previously described[2], were also amplified, and their sequences included in the study (Table 1). PCR products were purified for sequencing using the QIAquick Gel Extraction Kit (Qiagen) or ExoSap-It kit (Affymetrix, Cleveland, OH, USA). Analytical sequencing was completed at the UT Genomics Core (Knoxville, TN, USA) and McLab (South San Francisco, CA, USA). Sequences were assembled and manually corrected with Lasergene suite (DNASTAR, Madison, WI, USA). MAFFT version 7 with default settings[26, 27] was used for alignment, and Mesquite version 3.2[28] for trimming at the primer and terminal regions with uncertainty to the base call. The resultant point mutations (in rare instances, indels) were analyzed for changes in restriction patterns using Lasergene suite, and confirmed using the gDNA samples of the *C*. *florida* and *C*. *kousa* cultivars used for sequencing before analyses of the whole collection. Retrieved sequences were deposited in GenBank: cpDNA01 as MG575343 through MG575367; cpDNA02 as MG575368 through MG575385; cpDNA03 as MG575386 through MG575410.

**Table 1:** Cultivars and accessions of *Cornus florida*, *C*. *kousa*, and other related *Cornus* species subjected to analytical sequencing of PCR products of cpDNA01, 02, and 03.

*Cornus florida* cultivars and accessions^a^	*Cornus kousa* cultivars^a^	Other *Cornus* species^b^
Jean’s Appalachian Snow (2)^c^ Appalachian Spring Cherokee Brave Cherokee Chief Rutgers H4AR15925	Blue Shadow (2) Greensleeves National Pam’s Mountain Bouquet (2) Satomi Wolfeyes	*C*. *alba* L. *C*. *alternifolia* L.f. *C*. *amomum* Mill. *C*. *capitate* Wall. Ex Roxb. *C*. *elliptica* (Pojark.) QY Wadlford *C*. *eydeana* QY Xiang & YM Shui *C*. *hesseyii* Koehne *C*. *hongkongensis* (Hemsl.) Hutch. *C*. *mas* L. *C*. *nuttallii* Audubon ex Torr. & A. Gray *C*. *officinalis* Torr. Ex Dur. (Seib. & Zucc.) *C*. *pumila* Koehne *C*. *racemosa* Ram. *C*. *sericea* L. *C*. *controversa* (Hemsl.)

^**a**^ Plant samples obtained from the UT collections (University of Tennessee, Knoxville, TN), Rutgers University (New Brunswick, NJ), and Commercial Nursery (Decherd, TN).

^**b**^ Samples used in previous study[24].

^c^ Biological replicates in parentheses, *e*.*g*., sequencing of PCR products for cpDNA01, 02, and 03 from independent gDNA samples.

### Estimating genetic differences between species and chlorotype networks

A simplified data set of chlorotypes with only unique cultivar/line entries was subjected to χ2 test (α = 0.05) for pair-wise comparisons of the analyzed groups. This included the published results of wild *C*. *florida* from Call et al.[18]; (*n* = 225), *C*. *florida* cultivars/breeding selections collection (*n* = 91), *C*. *kousa* collection (*n* = 109), and other *Cornus* spp. group (*n* = 32). To infer the partition of the discovered variation, we used an AMOVA with MonteCarlo test of 999 repeats, an established method incorporating genetic distance measure based on genotype agreement, run with R version 3.4.0 and package *poppr*[29–31]. Separate analyses were executed on the generated chlorotyping data using the restriction sites of Call et al.[18], and also on data that included the newly discovered restriction sites.

The following algorithm was used on the sequences of cpDNA 01, 02, and 03, to infer their phylogenetic relationships. The MAFFT-aligned sequence matrices of cpDNA01, 02, and 03 of *C*. *florida* accessions, *C*. *kousa* cultivars, and of other *Cornus* species were trimmed using SeaView G block module to remove the uninformative sites with all options for the less stringent selection, and concatenated using Sequence Matrix[32]. Both separate and concatenated sequence matrices were analyzed using the Maximum-Likelihood Black Box (RAxML[33] with 100 repetitions and a bootstrap of 10,000; ML with thorough bootstrap), setting all the Blue-/White-Fruited dogwoods sequences or all the non-kousa/non-florida Big-Bracted dogwoods as a multiple outgroup, respectively. The RAxML used the ML algorithm with the molecular substitution matrix of GTR and the rapid hill-climbing mode. The matrix of concatenated sequences without G block curation was also visualized using mVista viewer[34]. This program used the AVID comparison module[35] against the *C*. *controversa* Hemsl. (syn. *Swida controversa*) concatenated sequence, to visualize the sequence similarity across the analyzed regions. This baseline sequence in turn originated from the alignment of the consensus cpDNA01, 02, and 03 of *C*. *florida* and *C*. *kousa* sequences obtained in this study, with the NCBI deposited complete cpDNA for this species (KU852492.1; NC_030260.1), using MAFFT with default settings. To compare our results with the publicly available resources, the NCBI database was mined using NCBI-BLAST and the retrieved sequences were pairwise MAFFT-aligned and submitted for similarity scoring using GUIDANCE-HoT with a default bootstrap of 100[36].

Genealogical relationships among the sequences were visualized using SplitsTree 4 version 4.14.5[37]. The following settings and assumptions were used: character transformation (uncorrected *P*); distance transformation (Neighbor Network); splits transformation (Equal Angle); splits post process (filter = dimension; value = 4; exclude = ‘no missing’); reticulate transformation (Reticulate Equal Angle). Chlorotype networks were inferred from the chlorotyping data set (four or 12 characters per individual) using R version 3.4.0 and package *poppr*[29–31], and visualized as the Minimum-Spanning Networks based on the Nei’s genetic distance[38, 39].

## Results

### Chlorotyping dogwoods

The gDNA samples of *C*. *florida*, *C*. *kousa*, and representatives of other *Cornus* species native to different geographical regions were compared with the recently published data of wild *C*. *florida* using their original chlorotyping protocol[18]. Chlorotypes of the *C*. *florida* accession collection from this study were distributed among the previously described four types. The majority of our specimens (including the commercial cultivars) had the H4 type described by Call et al.[18] (Table 2). All *C*. *kousa* samples exhibited the H3 type, both in the cultivars/breeding selections collection, and in samples of the Asia-originating wild accessions. In the *Cornus* spp. group, the majority of the samples displayed a previously undescribed chlorotype with the H3 type ranked as the second most abundant subset (Table 2).

**Table 2:** Frequencies of chlorotypes from four and 12 analytic sites among the tested dogwood (*Cornus* species) groups. Each row denotes one cpDNA chlorotype (sequence of restriction digest results).

Chlorotype^a^	Wild *C florida*[18]^b^	*C*. *florida* cultivars	*C*. *kousa* cultivars	other *Cornus* species
**4 sites [% frequencies]**				
0001	1	-	-	-
0011 **[2]**^**c**^	17	1	-	-
0101 **[1]**	46	13	-	-
0111 **[4]**	24	82	-	6
1100	1	-	-	-
1101	-	-	-	66
1110 **[3]**	7	3	100	-
1111	-	-	-	28
**12 sites [% frequencies]**				
001100111111	ND^d^	1	-	ND
010100111111	ND	12	-	ND
010101111111	ND	1	-	ND
011100011111	ND	1	-	ND
011100111101	ND	1	-	ND
011100111111	ND	80	-	ND
111010111111	ND	3	-	ND
111010000000	ND	-	2	ND
111010000111	ND	-	1	ND
111011000000	ND	-	51	ND
111011000001	ND	-	19	ND
111011001010	ND	-	1	ND
111011010000	ND	-	13	ND
111011010001	ND	-	10	ND
111011110000	ND	-	2	ND
111011111111	ND	-	1	ND

^a^ Digestion patterns (0 = no digest; 1 = digest), in the following order: (4 sites) cpDNA01d*AcuI*; cpDNA02d*TaqI*; cpDNA02d*Tsp45I*; cpDNA03d*SwaI*; extended in (12 sites) cpDNA01d*Hpy188I*; cpDNA01d*MseI*; cpDNA01d*MspI*; cpDNA01d*TaqI*; cpDNA02d*Hpy188I*; cpDNA03d*AluI*; cpDNA03d*ApoI*; cpDNA03d*MmeI*.

^b^ Group counts: wild *C*. *florida* (*n* = 225); *C*. *florida* cultivar collection (*n* = 91); *C*. *kousa* cultivar collection (*n* = 109); other *Cornus* spp. group (*n* = 32).

^c^ **[Bracketed bolded numbers]** under (4 sites) indicate the cpDNA chlorotypes as per Call et al.[18].

^d^ ND: Not determined by Call et al.[18], or not specified due to complex digestion pattern (*Cornus* spp. group).

Statistical analyses using Pearson’s χ^2^ test and AMOVA indicated significant (*P* < 0.001) differences among the four groups (Table 3; *C*. *florida* cultivars; *C*. *kousa* cultivars; wild *C*. *florida*; other *Cornus* species). Additionally, the within-groups diversity contributed almost twice as much to the variance as the between groups diversity in this analysis. Similarly, significant differences (Pearson’s χ^2^ *P* < 0.001) were detected between the *C*. *florida* group and the wild *C*. *florida* dataset (AMOVA: within groups: 15.1%, between groups: 84.9%). The same (Pearson’s χ^2^ *P* < 0.001) was true for the comparison between the *C*. *florida* group and the *C*. *kousa* group largely due to all the *C*. *kousa* samples exhibiting the H3 type. The within-groups diversity was then analyzed separately with regards to each group’s contribution. Here, the overwhelmingly largest contribution was attributed to the most abundant wild *C*. *florida* group, regardless whether it was compared among the four groups or only compared with the *C*. *florida* collection (Table 3).

**Table 3:** Results for AMOVA test of the binary dataset of *Cornus* species chlorotypes considering cpDNA01, 02, and 03 regions.

	Groups compared	Group count (*n*)	AMOVA		
			% variation	Significance	Within-group variation [%]
**4 sites**	wild *C*. *florida*[18]	225	Between: 36.1	*P* < 0.001	82
	*C*. *florida* cultivars	91			11
	*C*. *kousa* cultivars	109	Within: 63.9		0
	other *Cornus* spp.	32			7
**4 sites**	wild *C*. *florida*[18]	225	Between: 84.9	*P* < 0.001	88
	*C*. *florida* cultivars	91	Within: 15.1		12
**12 sites**	*C*. *florida* cultivars	91	Between: 8.6	*P* < 0.001	71
	*C*. *kousa* cultivars	109	Within: 91.4		29

### New chlorotyping sites

**Fig 1:**
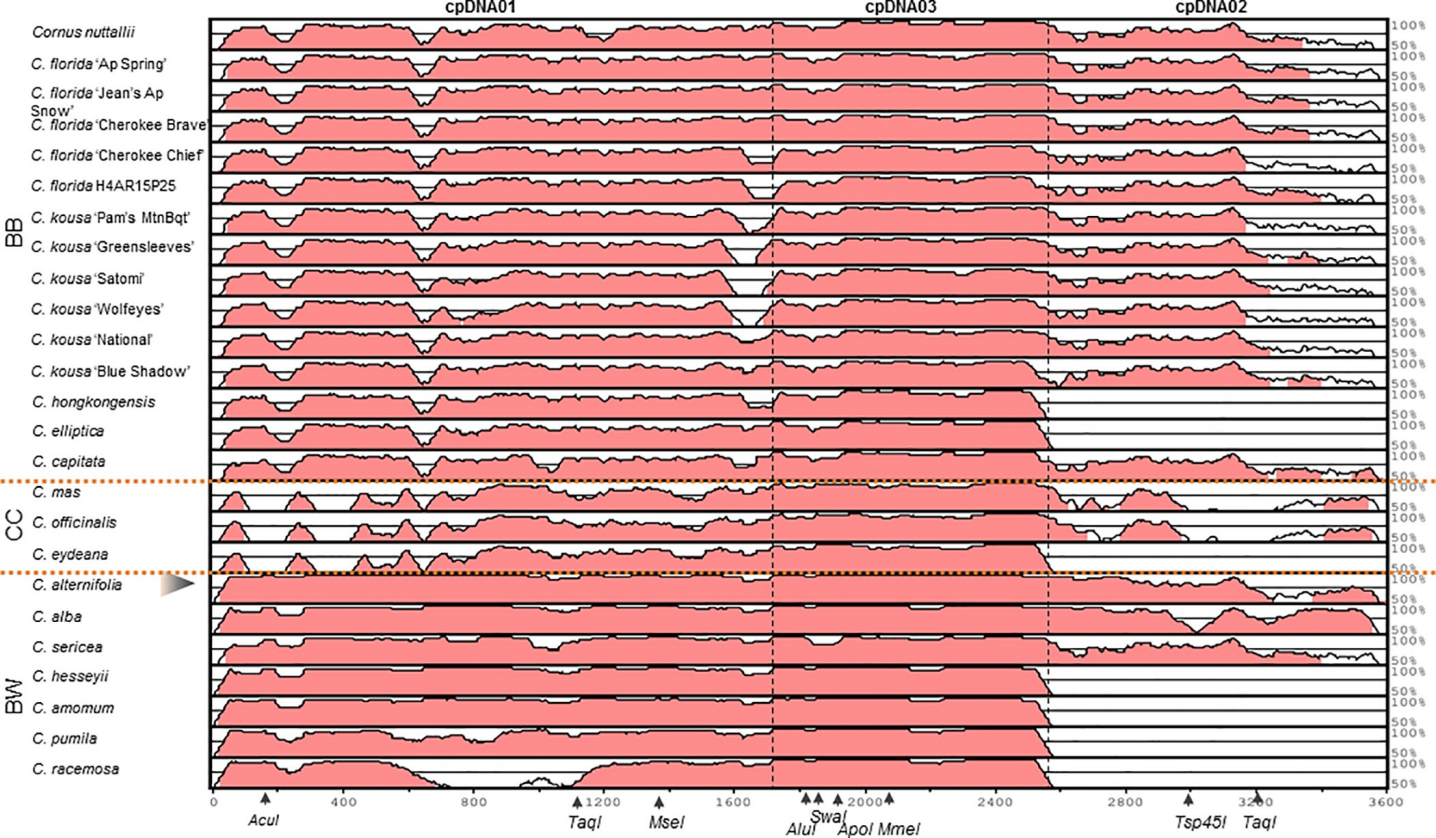
Sequence variation across the concatenated cpDNA01, 02, and 03 of dogwoods. mVISTA-based identity plots showing sequence similarity (%; height of the graphs) between the 25 sequenced accessions of *Cornus* (described on the left) in relation to the respective concatenated fragments of the reference sequence of *Cornus controversa* (KU852492.1; NC_030260.1). Concatenation was done in the order cpDNA01-03-02 (labeled on top), with the order having no impact on the subsequent analyses. Red shading of the mVista viewer denotes sequences recognized as non-coding; white shading denotes sequences unrecognized in character. Vertical dashed lines denote borders between the cpDNA regions analyzed. Horizontal dotted lines reflect the genus grouping as in [2] (BB: Big-Bracted dogwoods; CC: Cornelian Cherries; BW: Blue-/White-Fruited dogwoods). Black arrowhead indicates the expected phylogenetic position of *C*. *controversa*. Approximate locations of informative mutation sites are indicated below the graph, with enzymes names. Digestions cpDNA01d*MspI*, cpDNA01d*Hpy188I*, and cpDNA02*Hpy188I* were based on multiple sites, and omitted here for clarity. Abbreviated cultivars names: Ap Spring (Appalachian Spring); Jean’s Ap Snow (Jean’s Appalachian Snow); Pam’s Mtn Bqt (Pam’s Mountain Bouquet). Two BB and five BW cpDNA02 sequences were missing due to primer looping during sequencing, detected as multiple peaks over a substantial part of the chromatogram, and were thus omitted.

Analyses of the sequencing results from five *C*. *florida* accessions and six *C*. *kousa* cultivars (Table 1) indicated the presence of previously unreported informative nucleotide substitutions within the cpDNA regions of focus. All three regions, cpDNA01, 02, and 03, contained additional analytically usable mutations, which were confirmed with the restriction digests and gel electrophoresis analyses. Over the three analyzed regions, eight new substitutions were discovered, extending the chlorotyping panel. Most of these sites were specific to a species (*e*.*g*., *C*. *florida* vs. *C*. *kousa*); the remaining added within-species resolution (*e*.*g*., the cpDNA03d*MmeI* for *C*. *kousa*). High variation within the *Cornus* spp. group was also discovered using the new chlorotyping sites, specifically cpDNA01d*TaqI*, cpDNA02d*Hpy188I*, and cpDNA03d*ApoI* (Fig 1; S1 Table).

Due to the absence of data for the new chlorotyping sites in the wild *C*. *florida* dataset and the complexity of additional digestion products in the *Cornus* spp. group, only the data of *C*. *florida* and *C*. *kousa* cultivars for all 12 chlorotyping sites were compared using Pearson’s χ2 test and AMOVA (Tables 2 and 3). Both analyses indicated significant differences between the two groups (Pearson’s χ2 *P* < 0.001). Moreover, the within-group variation accounted for over 91% of the total variation. When the within-group variation between the collections of *C*. *florida* and C. *kousa* was analyzed for each group’s contribution, 71% of variation originated in the comparatively less abundant *C*. *florida* collection (Table 3). Over the 12 informative sites in cpDNA01, 02, and 03, seven newly defined chlorotypes for the *C*. *florida* collection were differentiated with eight restriction sites and nine chlorotypes in the *C*. *kousa* collection at seven restriction sites. None of the chlorotypes was shared between these two groups (Table 2).

### Phylogeny and network analyses

The sequences of cpDNA01, 02, and 03 for the five *C*. *florida* accessions, six *C*. *kousa* accessions, and 14 of the other *Cornus* species (Table 1) were aligned for phylogenetic analyses. Ratio of informative (variable) sites of each of the region differed per species (*C*. *florida* vs. *C*. *kousa*), group (Big-Bracted, Cornelian Cherries, Blue-/White-Fruited dogwoods), as well as per single region vs. concatenated sequences used (S3 Table). In particular, the proportion of variable sites differed for cpDNA01 between *C*. *florida* and *C*. *kousa* sequences. Mining of the NCBI library with the consensus sequences of cpDNA01, 02, and 03 for *C*. *florida* and *C*. *kousa* resulted only in the hits for these regions from *C*. *controversa* (KU852492.1 *S*. *controversa* voucher SCONT20150712 chloroplast, complete genome, 158,674 bp), and was included in the subsequent analyses. Only a few other *Cornus* sequences of focus were available, all of them for cpDNA03, and included the following: *C*. *nuttallii* (FJ541998.1), *C*. *mas* (AJ430988.1), *C*. *florida* (GQ998132.1), and two incomplete sequences of *C*. *kousa* (AB237418.1 and AB237417.1). All showed very high to perfect similarity index values to the sequences obtained from specimens included in this study (S1 Fig).

**Fig 2:**
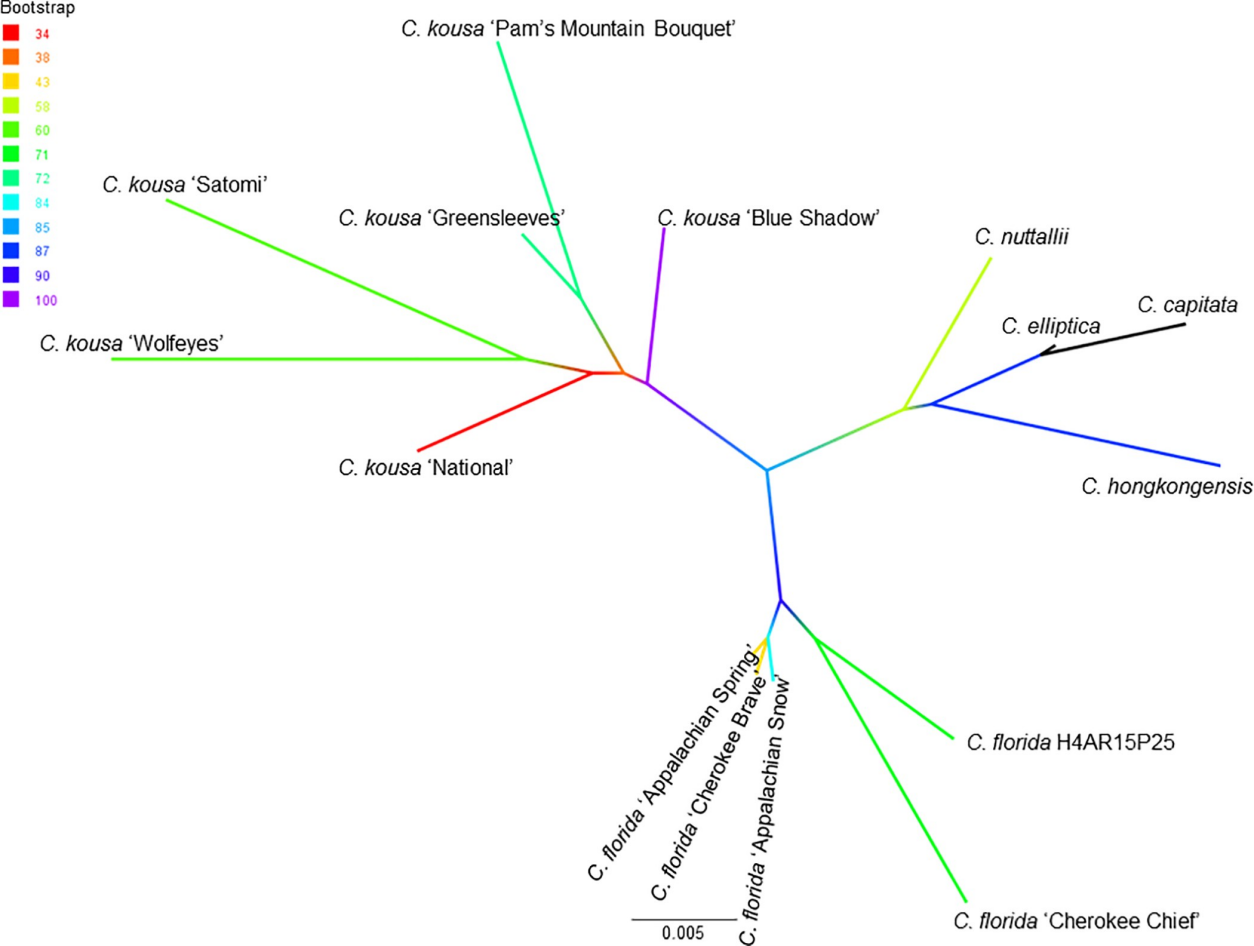
Best Maximum-Likelihood tree on concatenated and G blocks-trimmed sequences of cpDNA01-03-02 of the *Cornus florida* and *C*. *kousa*. The tree was constructed using RAxML Black Box, with 100 runs and a bootstrap of 10,000. The bootstrap support values are color-coded for each branch, and described on the accompanying legend. Concatenated sequences of the Big-Bracted dogwood species used in this study (*C*. *capitata*, *C*. *elliptica*, *C*. *hongkongensis*, and *C*. *nuttallii*) served as multiple outgroup.

**Fig 3:**
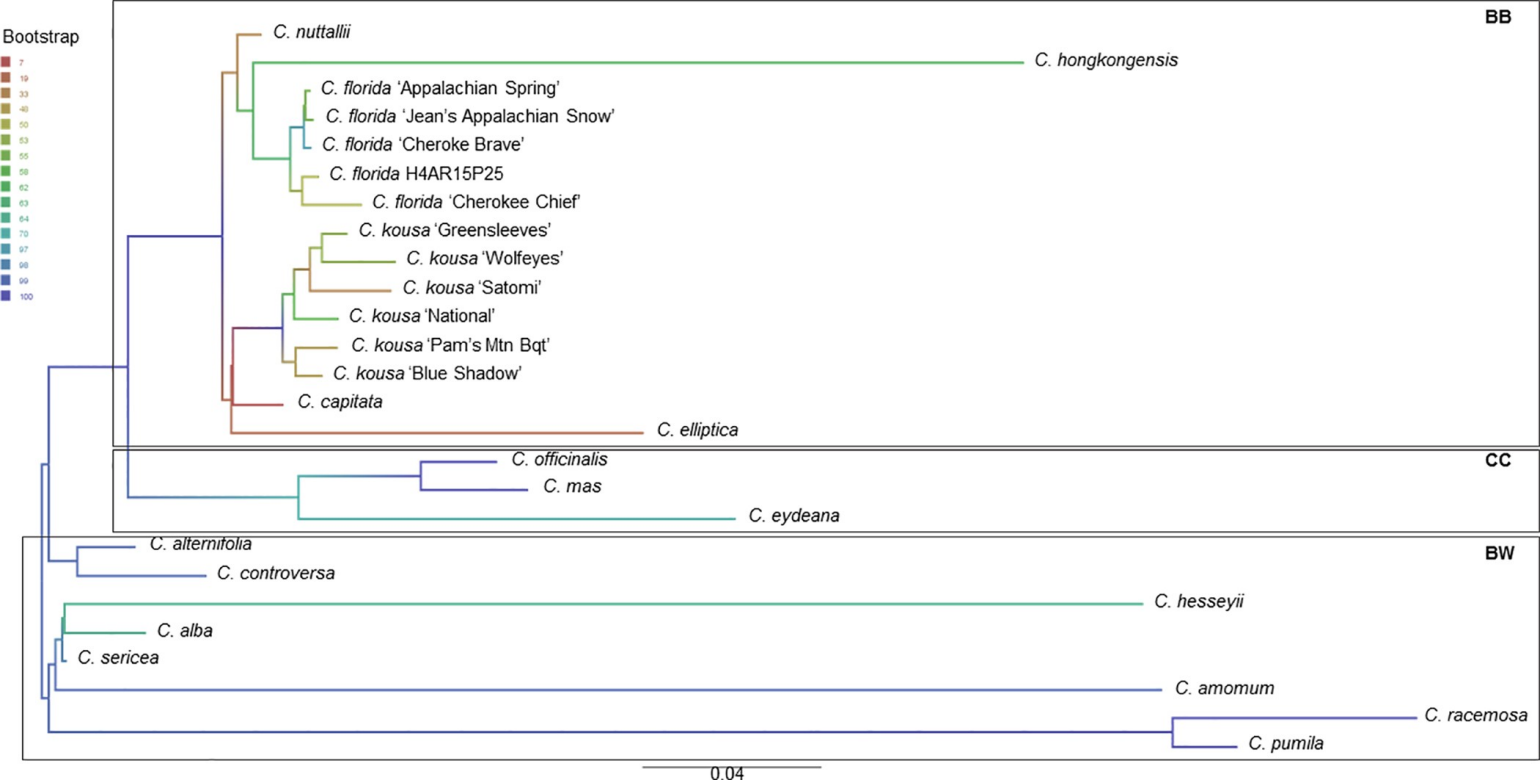
Best Maximum-Likelihood tree on concatenated and G blocks trimmed sequences of *Cornus* species cpDNA01-02-03. The tree was constructed using RAxML, with 100 runs and a bootstrap of 10,000, and the BW sequences as outgroup. Due to heavy looping of the sequencing with one of the primers of cpDNA02, not all sequences could be fully read (*n* = 7; see Fig 1), and were thus excluded from analysis, necessitating the partitioned-model. Branch colors indicate the bootstrap support values, with numerical legend on the left. *C*. *controversa* Hemsl. sequences resulted from the alignment of the consensus sequences of cpDNA01, 02, and 03 of *C*. *florida* and *C*. *kousa* with the NCBI deposited complete cpDNA for this species (KU852492.1; NC_030260.1). BB = Big-Bracted dogwoods; BW = Blue-/White-Fruited dogwoods; CC = Cornelian Cherries, as grouped in [2].

We analyzed each of the tested cpDNA regions separately (S2 Fig), as well as after concatenating the obtained sequences (Figs 1, 2 and 3). The phylogenetic analyses of the latter employed the partition model to reflect both the concatenation, and the missing two cpDNA02 sequences from the Big-Bracted species (*C*. *elliptica* and *C*. *hongkongensis*), and five cpDNA02 sequences from the outgroup of *Cornus* spp. (Blue-/White-Fruited dogwoods; *C*. *amomum*, *C*. *eydeana*, *C*. *hessei*, *C*. *pumila*, *C*. *racemosa*; Fig 1). Missing data were not fully resolved due to looping with one of the primers used (*ndhF*; S2 Table), and were excluded from concatenated sequences (treated as ‘missing’).

The concatenated sequences of *C*. *florida* and *C*. *kousa* samples resolved very well with the Maximum-Likelihood (ML) approach (Fig 2). The best ML tree was based on the concatenated sequences of 3492±19 bp with the MAFFT alignment of 3524 characters over 354 distinct alignment patterns. The proportion of gaps and completely undetermined characters (ambiguous sites treated as Ns by RAxML, such as gaps introduced by multiple sequence alignment) was 4.6%.

The concatenated 26 sequences of *Cornus* cpDNA01, 02, and 03 were 3484±71 bp long, whereas the alignment was 3996 characters long with proportion of gaps and completely undetermined characters of about 12.4%. The *Cornus* genus ML tree from 781 distinct alignment patterns analysis (Fig 3) reflected well the taxonomic relationships within the genus. When comparing the ML trees from separate and concatenated sequences, only rarely were the changes in the phylogenetic order observed (Fig 3, S2 Fig). The affected sequences belonged mainly to the outgroup of Blue-/White-Fruited dogwoods and these position changes were attributed to incomplete sorting and/or the missing data from cpDNA02.

**Fig 4:**
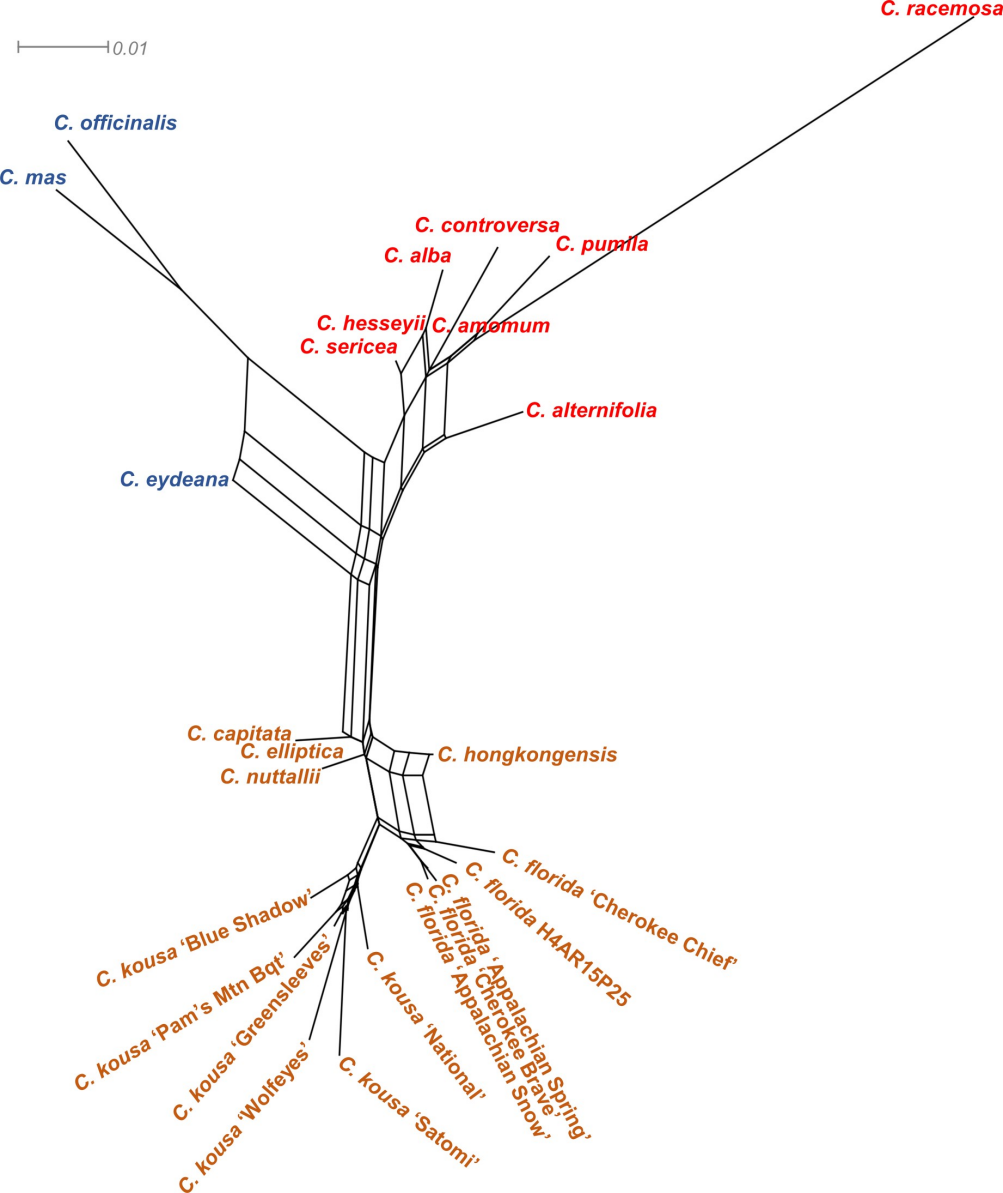
Genealogical relationships among *Cornus* species inferred from analyses of cpDNA01, 02, and 03 sequences using SplitsTree4. Big-Bracted dogwoods nameplates are in brown; Cornelian Cherries in blue; Blue-/White-Fruited dogwoods in red.

Visualization of the genealogical relationships in networks based on the sequences (SplitsTree; Fig 4) was in agreement with the relative grouping of the *Cornus* species by RAxML. Changes in the relative placement only appeared when each cpDNA region of focus were analyzed separately using this method (data not shown).

**Fig 5:**
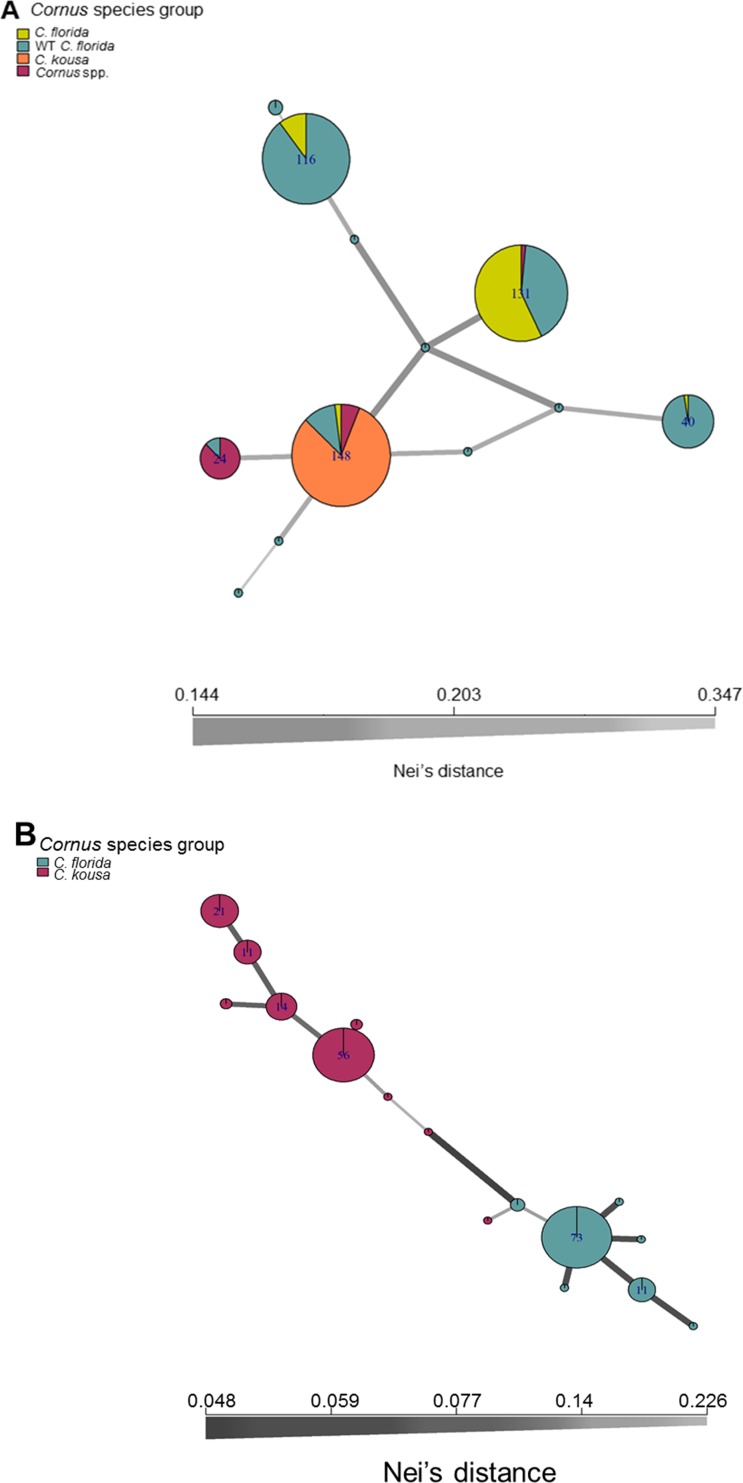
**Dogwood chlorotype network based on four (A) and 12 (B) informative mutations (restriction patterns) over cpDNA01, 02, and 03.** The Minimum Spanning Network of the chlorotypes was calculated and visualized in R with package *poppr* using Nei’s genetic distance[38, 39]. Nodes sizes reflect the number (frequency) of a given chlorotype, with nodes colors coding for groups used in the analysis as per respective figure legends. Edge widths and shading are reversely proportional to the Nei’s genetic distances between the respective nodes and are denoted on the corresponding legend.

Analyses of the chlorotype networks based on four or 12 characters (*i*.*e*., restriction digests; Fig 5A and 5B, respectively) suggested the tentative placement of the H3 type *sensu* Call et al.[18] as the most ancestral within the collection analyzed. This chlorotype showed the highest Eigenvector centrality values with the four restriction digests analyses that lacked the within-kousa resolution (Fig 5A). The same analysis highlighted the particularly broad placement of the wild accessions of *C*. *florida*, underlining the species rich diversity over the regions studied. In contrast, only the 12-character chlorotypes allowed insight into the *C*. *kousa* networks (Fig 5B), pointing towards only limited usefulness of the system based on the closely related *C*. *florida*.

## Discussion

In this study, we aimed to expand the chlorotyping strategy on the cpDNA of the two Big-Bracted dogwood collections, which extended the recent study in this area by Call et al.[18]. While doing so, we also used an economically attractive classical Sanger sequencing approach, alternative to a typical GBS, to improve the chlorotyping panel published therein. Use of the cpDNA for phylogenetic and phylogeographic studies, based on its particular advantages (*i*.*e*., uniparental inheritance, haploidy, very rare recombination, content and size stability) has contributed significantly to the achievements in plant biology over last 20 years[40–42]. Next-generation sequencing added to the momentum with comparisons of the complete cpDNA sequences[43–45]. This new vantage point confirmed the existence of evolutionary hot-spots, particularly in the non-coding cpDNA regions[19, 20, 46, 47].

Extensive research also revealed the limitations of using cpDNA at the lowest taxonomic ranks. For instance, in many studies, the genetic variability, evolutionary rate, and heterogeneity among lineages were not sufficient to define species at the lowest taxonomic ranks[48–50]. Moreover, despite the tremendous output of next-generation sequencing, the results sometimes yielded very limited differences among specimens of the same species. For example, McPherson et al.[45] reported that the seven single-nucleotide polymorphisms (SNPs) over the entire chloroplast genome of *Toona ciliata* M. Roem. only yielded six chlorotypes. Another issue to be addressed is the diversity of a given region respective to the intended research aim (*i*.*e*., taxonomic rank). Despite cpDNA showing generally lower variability than nuclear DNA, regions able to discriminate at species, subclade, or even as high as clade level were documented[44, 47–49].

At the species level, our approach seems to pose an economically attractive alternative to the whole-cpDNA genome sequencing, especially when the regions with adequate nucleotide variability are known[44, 47, 48]. Such variability was displayed in both coding and non-coding cpDNA regions alike. Among the coding cpDNA sequences, the *matK* and *ndhF* regions have been well-studied for species-level population genetics as well species relationships within a genus or deep phylogeny studies[2, 47, 48, 50, 51]. Since the start of the molecular cpDNA research dubbed “the tortoise and the hare race” to reflect the mutational pace differences in the coding vs. non-coding regions[51], and especially with its subsequent developments[19, 20, 50], the current body of research evidence has been assembled from several non-coding regions. Among these, the regions used in our study *ndhF-rpl32* and *trnQ-rps16* (with the partially overlapping *rps16* intron[52]) appeared very often with varying, but overall high research value for resolving species relationships within a genus attributed to them[20, 43, 44, 46, 48, 49]. The recent establishment of the cpDNA chlorotyping system for *C*. *florida* and the ensuing phylogenetic analyses of the *Cornus* genus[18, 53] is yet another confirmation of usefulness of these regions at lower taxonomic ranks. The usefulness of the chlorotyping system for *C*. *florida* diversity and evolution study[18] is further underscored and extended to other *Cornus* species in our follow-up research. This was achieved with major monetary savings in our double-directional Sanger sequencing of the PCR products of three cpDNA regions in 11 cultivars, *vs*. only partial coverage from the high-throughput transcriptome sequencing (HiSeq). Indeed, comparisons of our results with the HiSeq results indicated that the conventional approach carried significant advantages over the HiSeq of *C*. *florida* and *C*. *kousa* bracts and of *C*. *florida* leaves (S3 Fig; Nowicki et al. unpublished data). For *C*. *florida* and *C*. *kousa*, the HiSeq resulted in the cumulative 67% (max = six reads per bp) and 83% coverage (max = five reads per bp) of the *C*. *controversa* whole cpDNA genome, respectively. The 64 discovered SNPs and INDELs (insertions/deletions) distinguished the *C*. *florida* and *C*. *kousa* cultivars analyzed (no SNPs/INDELs between the two *C*. *kousa* cultivars; 10 SNPs/INDELs among the *C*. *florida* cultivars). All of them were located in the non-coding regions and grouped in three “hot spots” (S3 Fig; Nowicki et al. unpublished data).

Application of the cpDNA chlorotyping system to a collection of cultivars of both dogwood species addresses several issues, including the lineage tracking, the chlorotyping system transfer to a related species, and the assessment of species diversity captured in the cultivars. In this regard, despite the confirmation of the *C*. *florida* chlorotyping working for *C*. *kousa*, the cpDNA regions and/or informative sites undergoing analyses must be adjusted for the latter species. All of the *C*. *kousa* samples included in this study showed exclusively the *C*. *florida* H3 chlorotype *sensu* Call et al.[18], regardless whether they originated from cultivars, kousa-based hybrids with other *Cornus* species, breeding selections, or wild accessions from Asia. The non-resolution of the *C*. *florida*-based chlorotyping system in the closely related *C*. *kousa* collection with confirmed nucleotide variability within the regions of interest suggests different sites undergoing mutations in the two closely related species. But, the limitation of our study was the low sample number of wild *C*. *kousa* accessions from the species native range, with comparatively higher counts of commercial cultivars and breeding selections than for the *C*. *florida* collection. We also observed the appearance of the previously unreported chlorotypes in other *Cornus* spp., particularly in the clades distant from this of *C*. *florida* (*i*.*e*., the Big-Bracted group). The relative analytical value of a given cpDNA region changed drastically even among the closely related species[43, 44], as also documented in our study (S3 Table). Our discovery prompted analyses of the cpDNA sequences in cultivars of both species that resulted in eight additional analytically informative nucleotide substitutions. Most of these new restriction sites were species specific, but one (cpDNA03d*MmeI*) uncovered the within-kousa variability. It is possible that additional useful sites can be retrieved by sequencing these regions in additional cultivars. Although not all available cultivars of both species were analyzed in this project, those that were included represented the majority of the materials available commercially. Moreover, multiple entries for many cultivars received from several sources confirmed the chlorotypes and were in agreement for each such represented cultivar, respectively. In addition, we utilized the extended chlorotyping system to confirm the lineage of several interspecific hybrids, whose parentage was published recently[16]. For instance, “Wonder Berry”, “Red Beauty”, and “Rutnut Red Pygmy” were all properly assigned the maternal parentage of *C*. *florida*. And, conversely, “Rosy Teacups” was also properly identified using our chlorotyping system as being of *C*. *kousa* maternal background (S1 Table).

Studies comparing genetic diversity in wild and cultivated forms can greatly benefit from the features of cpDNA. A consensus conclusion emerging from many such studies indicates that cpDNA diversity found in wild forms exceeded the diversity exhibited in cultivated accessions. This was the case for *Prunus* cherries analyzed with five cpDNA markers[54], a *Musa* spp. collection analyzed with four non-coding cpDNA markers[55], and Mediterranean olive trees (*Olea europaea*, Oleaceae) with *de novo* sequencing of cpDNA[56]. In another striking example, a *Malus* spp. analyses used only one diverse cpDNA coding region, *mat*K, and two non-coding regions[57] yet yielded significant results. Sixteen chlorotypes were identified within their materials pool using this approach that allowed to estimate the genetic distances between wild and domesticated *Malus* species from various origins, and to identify hybrids and feral cultivars[57].

Research on *C*. *nuttallii* (Pacific dogwood), closely related to *C*. *florida*, combined the use of both nrDNA satellite regions and cpDNA regions[58, 59]. From the seven cpDNA markers used therein, only cpDNA03 (*rps16* intron) overlapped with our study. In the *C*. *nuttallii* studies, only two chlorotypes were identified with polymorphism in the *rpL16B* region, whereas five of the eight nrDNA microsatellite markers used had polymorphism useful for evaluating the species diversity. Regardless of the markers used, the overall species diversity was assessed as very low. These results again underline the need for careful choice of regions for chlorotyping analyses.

Very little molecular information has been deposited in GenBank on *Cornus* cpDNA overlapping with our research except for the recently deposited complete cpDNA sequence of *C*. *controversa*. The cpDNA01, 02, and 03 reported here represent just over 10% of the *C*. *controversa* non-coding cpDNA regions or about 2.3% of its complete cpDNA (S3 Fig; Nowicki et al. unpublished data). This coverage will increase with our continued development of a chlorotyping system for both Big-Bracted dogwoods using other popular non-coding cpDNA regions.

Our sequencing results indicated an appreciable variability over the three cpDNA regions. This suggested that the appropriate choice of regions allows for species-level analysis, but also for helping decipher the relationships of species within *Cornus*[2]. The cpDNA regions used in this analysis proved their usefulness and informativeness, similar to several other studies[44, 47, 48]. But, we discovered and postulated the need for more cpDNA regions/haplotyping of *C*. *kousa* to allow its population genetics and species diversity studies, as well as to improve its phylogenetic placement within the genus. The phylogenetic trees and genealogy networks constructed with the sequences obtained are in agreement with previous studies of the *Cornus* genus using the nuclear and extranuclear sequences alike[2, 21, 22, 60]. The exact placement of *C*. *controversa* and *C*. *alternifolia* among the other *Cornus* species requires additional research[22, 60]. Our on-going work with more cpDNA regions will likely improve the delineation of the species within the genus, especially using concatenated sequences for building a phylogeny. This approach is similar to the improved resolution of the cpDNA coding sequences upon concatenation[61, 62].

As an alternative to the system presented here, several studies used Simple Sequence Repeats (SSR) or microsatellite markers for analyses of cpDNA diversity. In the extreme case, as many as over 200 cpDNA SSR markers were scored[63]. Analyses of *C*. *controversa* complete cpDNA discourage the development of dinucleotide or higher SSR markers, as in total only two perfect [(AT)8; (TTA)5] and nine imperfect and compound repeats SSRs were identified (S4 Table). These estimates are low, but are to be updated when the *C*. *florida* genome work is completed (currently in progress).

The nuclear SSR markers have been developed for *C*. *florida* and *C*. *kousa*, and used to infer the levels of genetic diversity and spatial distribution in the native range of *C*. *florida*[9, 10, 64–66]. Comparatively, the *C*. *kousa* diversity is much less known. Only a few studies analyzed the species in this regard, mostly utilizing the phenotypic data, as part of the Korean conservation efforts[67]. A small study used six ISSR primers over 13 *C*. *kousa* wild accessions successfully distinguishing the red- and white-bracted ones, including their origin (the Korea mainland or Oenaro Island; ref. [68]). Cultivars of both *Cornus* species were studied in regard to their utility traits displayed, including pathogen resistance[11, 12] or anthocyanins production[5, 6]. In this regard, chlorotyping of the collection *C*. *kousa* cultivars, breeding selections, and wild Asian accessions is a novelty in several aspects. Our study showed that the *C*. *florida*-based chlorotyping system[18] did not differentiate any of the *C*. *kousa* samples, indicating differences in the nucleotide substitutions between the two species. But, extension of the chlorotyping panel by sequencing of the cpDNA regions of interest uncovered some analytically informative substitutions. Recent transcriptome study of 10 *Cornus* species confirmed a Cretaceous whole genome duplication event that coincided with the split of the three major lineages, after which, the rates of molecular evolution occurred independently in the various species[69]. In addition, phylogenetic analyses of the *Cornus* genus have been conducted using cpDNA sequences of *rbcL*, *matK*, *ndhF*, and *atpB* regions, as well as nuclear 26SrDNA, *antR-Cor*, and internal transcribed region (ITS)[2, 21–23]. These studies identified major lineages and resolved relationships among them. But, species relationships within the Big-Bracted group and the Blue-/White-Fruited group remained incompletely resolved due to incomplete sampling or insufficient informative phylogenetic characters from the molecular regions applied. Thus, our study suggested that the three cpDNA non-coding regions are potentially useful for resolving relationships within these dogwood groups due to increased number of informative sites in these cpDNA regions.

## Conclusions and outlook

The presented chlorotyping panel for *C*. *florida* and *C*. *kousa* suggests that their diversity has been captured in the cultivated forms at significant levels. Species diversity in the commercial cultivars measured with chlorotyping was lower than in the collection of wild *C*. *florida* accessions and suggests the species diversity present in nature will be useful for breeding. Similar comparisons for *C*. *kousa* dogwood accessions and cultivars can be completed using the chlorotyping protocols described in this study. We were able to improve on the previous *Cornus* chlorotyping research by sequencing only five *C*. *florida* and six *C*. *kousa* accessions over the three non-coding cpDNA regions underscoring the efficiency offered by our approach. Although the *C*. *florida* genome sequence is not yet available, our study adds sequence resources to the genus cpDNA information. Furthermore, our data suggests the *C*. *florida*-based H3 chlorotype as the most ancestral among the species tested, and underscores the need for testing further regions in regards of their usefulness for *Cornus* species delineation within the genus and molecular diversity.

## Supporting information

S1 Fig Available NCBI-deposited sequences of cpDNA03 from the *Cornus* genus align very well with those obtained in our study. Respective Genbank numbers and GUIDANCE (HoT) alignment scores are indicated for the sequences of each species analyzed: (A) *C*. *florida* (1,000000); (B) *C*. *kousa* (0,992379); (C) *C*. *mas* (0,999432); (D) *C*. *nuttallii* (1,000000). Sequences obtained in the course of this study are appended with “UTK”.
(TIF)

S2 Fig **Best Maximum-Likelihood phylogenetic trees of the MAFFT-aligned sequences from cpDNA01 (A), cpDNA02 (B), and cpDNA03 (C) obtained in this study.** Sequences were G regions trimmed (SeaView; low stringency) after MAFFT alignment to remove the uninformative characters. The RAxML settings of 100 runs and a bootstrap of 10,000 were used. The sequences of Blue-/White-Fruited group served as multiple outgroup. Colored edges indicate the bootstrap support values, with the numerical legends accompanying, respectively. The cpDNA01 alignment was 1667 characters long, and the tree was produced by running 391 alignments, with 3.6% of gaps and completely undetermined characters. The cpDNA02 alignment was 986 characters long, and the tree was produced by running 283 alignments, with 1.6% of gaps and completely undetermined characters. Due to heavy primer looping in the other *Cornus* species group, seven sequences were omitted (see Figs 1 and 3 in the main text). The cpDNA03 alignment was 907 characters long, and the tree was produced by running 82 alignments, with 1.6% of gaps and completely undetermined characters.
(TIF)

S3 Fig Coverage and polymorphisms retrieved by high-throughput transcriptome sequencing (HiSeq) of *Cornus florida* bracts and leaves and *C*. *kousa* bracts. Total RNA extracted from the bracts of *C*. *florida* (*n* = 8; three unique cultivars), bracts of *C*. *kousa* (*n* = 7; two unique cultivars), and leaves of *C*. *florida* (*n* = 8; four unique cultivars) were submitted for commercial Illumina HiSeq (GeneWiz, South Plainfield, NJ, USA). Reads produced by the Illumina HiSeq system were subjected to error correction using Rcorrector (RNA-Seq error CORRECTOR)[70]. Next, sequencing adapters were trimmed off and short reads (<30 bases) were excluded from RNA-Seq analysis using the Skewer program version 0.2.2[71]. Read quality control was performed using FastQC version 0.11.4[72]. The resulting fragments were mapped to a *C*. *florida* or *C*. *kousa* transcriptome, respectively[69], using GSNAP version 2018-01-31[73]. The assemblies were aligned to the chloroplast DNA samples obtained from *C*. *controversa* (NCBI NC_030260.1 and MG525004.1, respectively) using the BBMap short read aligner with default parameters[74]. This determined the overall alignment of the query *Cornus* species to the cpDNA; the samtools depth command was used to take this information and determine how many times a query read matched to a reference base[75]. Once complete, the data was stored into a text file containing the cpDNA reference base and locus, and how many times a query read was found to map with it. In addition to its short read aligner, BBMap contains a number of other tools for analysis of bioinformatics data. Of particular interest to this study is its variant calling command, which takes one or more SAM/BAM files, compares it to a reference Fasta file, and identifies single nucleotide polymorphisms (SNPs), insertions/deletions (INDELs) in the query files. Using the SAM files generated by BBMap to generate depth coverage, this command was used to call variants from the *C*. *florida* data, the *C*. *kousa* data, and from both species, compared to the *C*. *controversa* cpDNA. The results were visualized using R version 3.4.4[31] package circlize version 0.4.4[76]. The most outer circle denotes the *C*. *controversa* cpDNA regions (LSC: large single-copy region; IRB: inverted repeat B; SSC: small single-copy region; IRA: inverted repeat A). The second tier marks (in light brown) the cumulative coverage of the baseline of NCBI NC_030260.1 and MG525004.1 by the *C*. *kousa* transcpritomes (max = 5); base pairs order as in the NC_030260.1. The third tier marks (in magenta) the SNPs and INDELs between *C*. *kousa* and *C*. *florida* transcriptomes mapped back to *C*. *controversa* NC_030260.1 and MG525004.1; no differences were discovered between the two *C*. *kousa* cultivars analyzed. The next tier marks (in grey) the coding (high) and non-coding (low) regions of *C*. *controversa* NC_030260.1. The next tier marks (in green) the SNPs and INDELs discovered in the transcriptomes of the various *C*. *florida* cultivars and accessions analyzed. The next tier marks (in orange) the cumulative coverage of the baseline of *C*. *controversa* NC_030260.1 MG525004.1 by the *C*. *florida* transcpritomes (max = 6). Finally, the innermost tier marks (in blue) the regions analyzed in detail in this paper, cpDNA 01, 02, and 03. The cpDNA01 and cpDNA 03 partially overlap.
(TIF)

S1 Table Detailed chlorotyping results of the simplified (unique entry) *Cornus* collection. Within groups (*C*. *florida* collection; *C*. *kousa* collection; other *Cornus* species collection), cultivars/lines/accessions were ordered alphabetically. Chlorotyping results as per Call et al.[18] are presented first, with the extended chlorotyping panel in the following columns. Respective region codes and restriction enzymes used are indicated in the column headers. Results are presented as 0s (no digest) and 1s (digest); more complex patterns are marked with characters other than 0s or 1s. “?” denotes inconclusive results.
(XLSX)

S2 Table Analytical details showing the cpDNA regions used in the study, their respective primers sequences, PCR conditions, and product sizes. (DOCX)

S3 Table Proportion of variable sites in the MAFFT-aligned sequences. Reported % values (in parentheses–base pair length of the alignment) for aligned cpDNA01, 02, 03, and concatenated sequences. Sequenced samples are listed out in Table 1. Sequences were aligned using MAFFT without G regions trimming of uninformative sites. *Cornus* species grouping as in ref. [2].
(DOCX)

S4 Table Simple sequence repeats (SSRs) detected in the cpDNA of *Cornus controversa* pose little alternative to chlorotyping. The SSRs were detected in the sequence (KU852492.1; NC_030260.1 voucher SCONT20150712 chloroplast, complete genome, 158,674 bp) using the https://ssr.nwisrl.ars.usda.gov with the default settings.
(DOCX)
