# Supplementary figures and images for: Haplotyping of Cornus florida and C. kousa chloroplasts: Insights into species-level differences and patterns of plastic DNA variation in cultivars
Source: PLoS One. 2018 Oct 23;13(10):e0205407. doi: 10.1371/journal.pone.0205407 (PMC6198962; doi:10.1371/journal.pone.0205407)

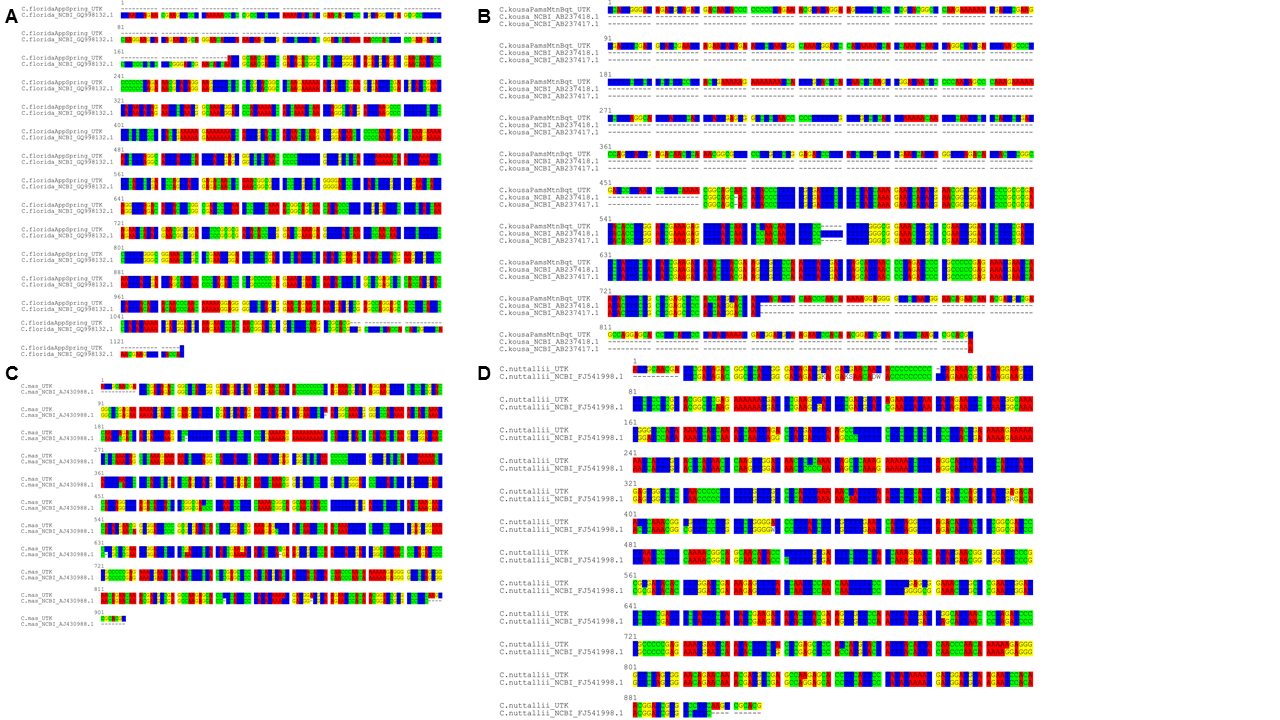

Supplement: S1 Fig — Respective Genbank numbers and GUIDANCE (HoT) alignment scores are indicated for the sequences of each species analyzed: (A) C. florida (1,000000); (B) C. kousa (0,992379); (C) C. mas (0,999432); (D) C. nuttallii (1,000000). Sequences obtained in the course of this study are appended with “UTK”. (TIF) [file pone.0205407.s001.tif]

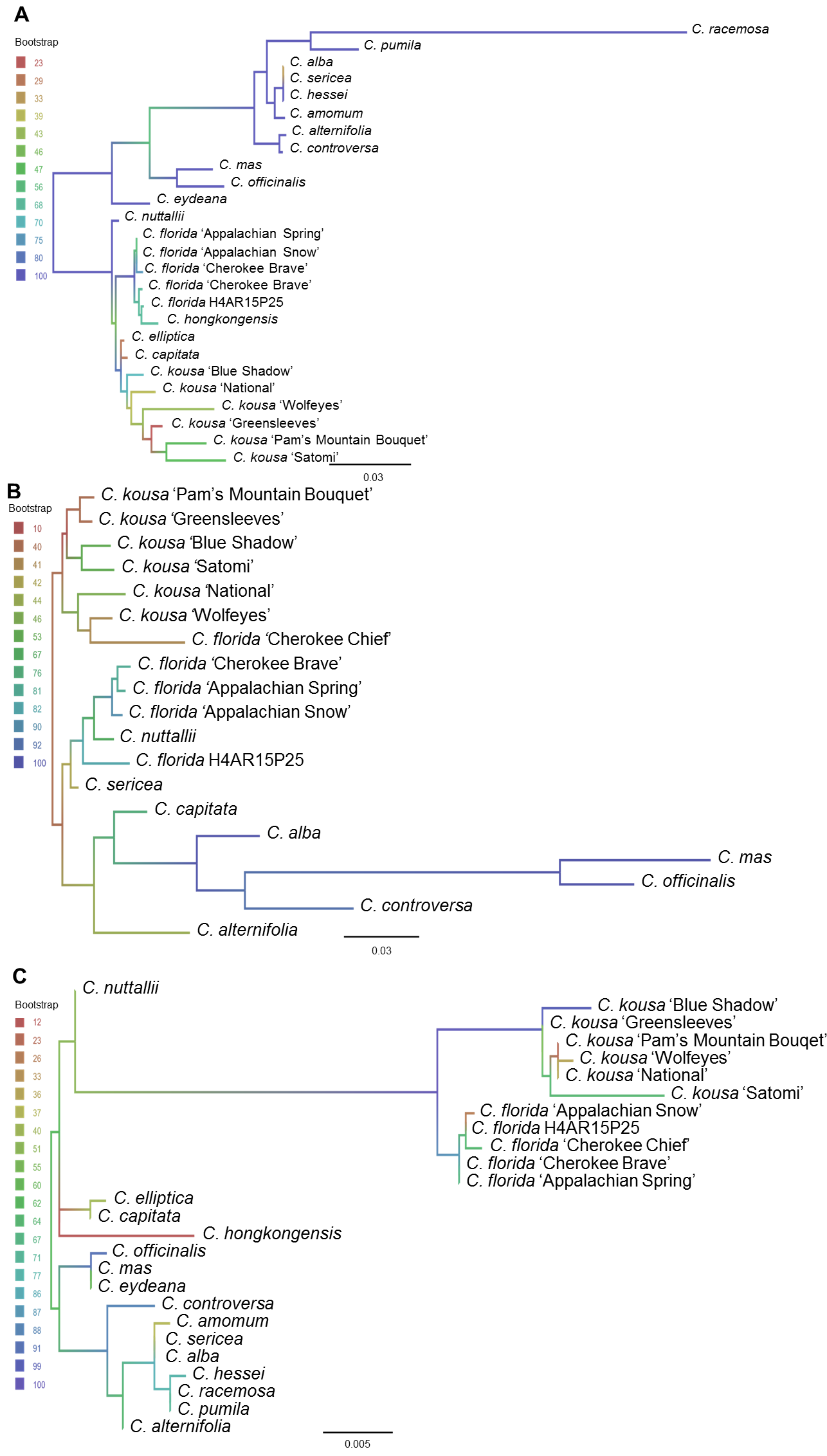

Supplement: S2 Fig — Best Maximum-Likelihood phylogenetic trees of the MAFFT-aligned sequences from cpDNA01 (A), cpDNA02 (B), and cpDNA03 (C) obtained in this study. Sequences were G regions trimmed (SeaView; low stringency) after MAFFT alignment to remove the uninformative characters. The RAxML settings of 100 runs and a bootstrap of 10,000 were used. The sequences of Blue-/White-Fruited group served as multiple outgroup. Colored edges indicate the bootstrap support values, with the numerical legends accompanying, respectively. The cpDNA01 alignment was 1667 characters long, and the tree was produced by running 391 alignments, with 3.6% of gaps and completely undetermined characters. The cpDNA02 alignment was 986 characters long, and the tree was produced by running 283 alignments, with 1.6% of gaps and completely undetermined characters. Due to heavy primer looping in the other Cornus species group, seven sequences were omitted (see Figs 1 and 3 in the main text). The cpDNA03 alignment was 907 characters long, and the tree was produced by running 82 alignments, with 1.6% of gaps and completely undetermined characters. (TIF) [file pone.0205407.s002.tif]

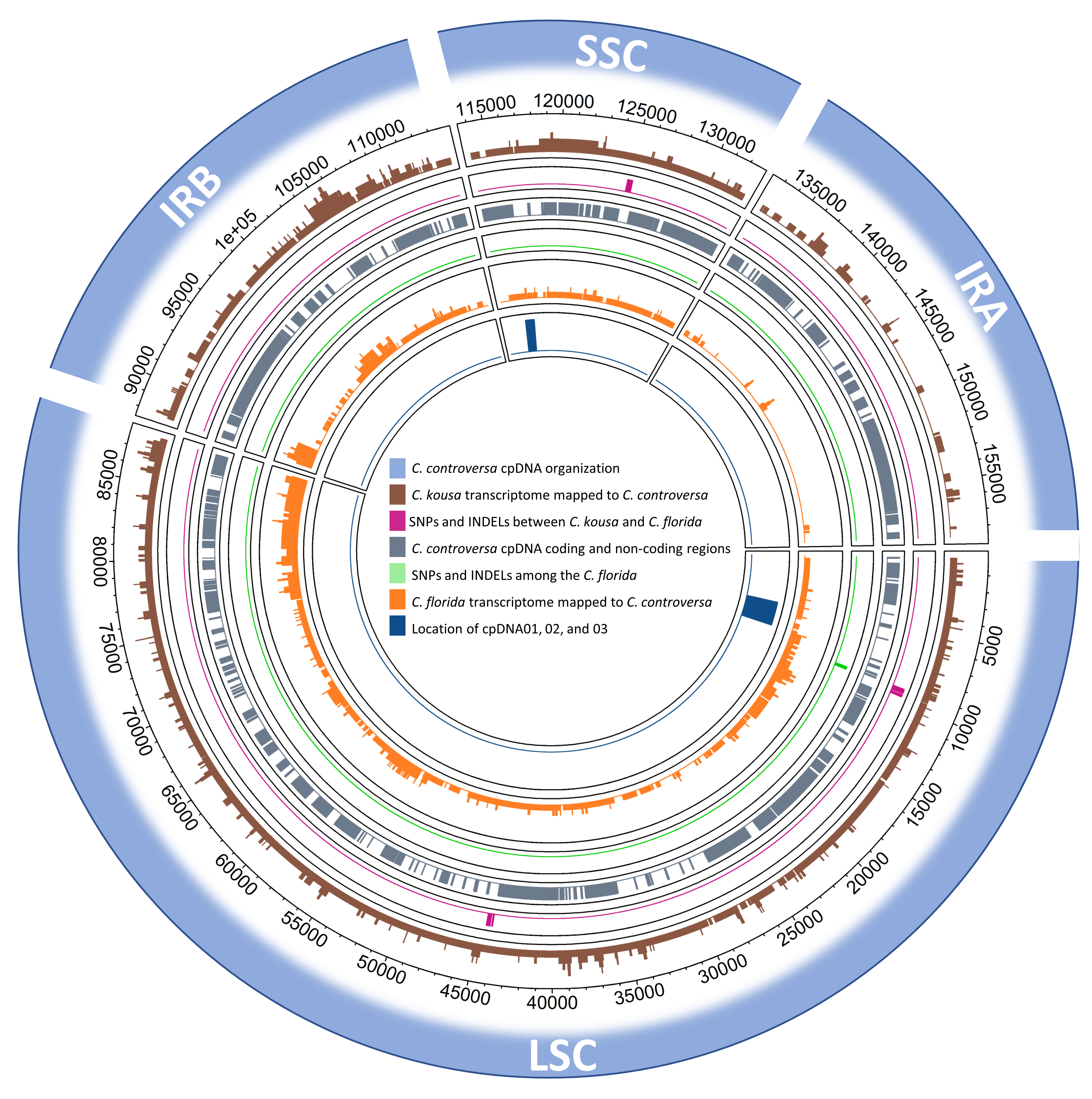

Supplement: S3 Fig — Total RNA extracted from the bracts of C. florida (n = 8; three unique cultivars), bracts of C. kousa (n = 7; two unique cultivars), and leaves of C. florida (n = 8; four unique cultivars) were submitted for commercial Illumina HiSeq (GeneWiz, South Plainfield, NJ, USA). Reads produced by the Illumina HiSeq system were subjected to error correction using Rcorrector (RNA-Seq error CORRECTOR)[70]. Next, sequencing adapters were trimmed off and short reads (<30 bases) were excluded from RNA-Seq analysis using the Skewer program version 0.2.2[71]. Read quality control was performed using FastQC version 0.11.4[72]. The resulting fragments were mapped to a C. florida or C. kousa transcriptome, respectively[69], using GSNAP version 2018-01-31[73]. The assemblies were aligned to the chloroplast DNA samples obtained from C. controversa (NCBI NC_030260.1 and MG525004.1, respectively) using the BBMap short read aligner with default parameters[74]. This determined the overall alignment of the query Cornus species to the cpDNA; the samtools depth command was used to take this information and determine how many times a query read matched to a reference base[75]. Once complete, the data was stored into a text file containing the cpDNA reference base and locus, and how many times a query read was found to map with it. In addition to its short read aligner, BBMap contains a number of other tools for analysis of bioinformatics data. Of particular interest to this study is its variant calling command, which takes one or more SAM/BAM files, compares it to a reference Fasta file, and identifies single nucleotide polymorphisms (SNPs), insertions/deletions (INDELs) in the query files. Using the SAM files generated by BBMap to generate depth coverage, this command was used to call variants from the C. florida data, the C. kousa data, and from both species, compared to the C. controversa cpDNA. The results were visualized using R version 3.4.4[31] package circlize version 0.4.4[76]. The mos [file pone.0205407.s003.tif]
