# Supplementary material for: Haplotyping of Cornus florida and C. kousa chloroplasts: Insights into species-level differences and patterns of plastic DNA variation in cultivars
Source: PLoS One. 2018 Oct 23;13(10):e0205407. doi: 10.1371/journal.pone.0205407 (PMC6198962; doi:10.1371/journal.pone.0205407)
Supplement: S2 Table — (DOCX) [file pone.0205407.s005.docx]

S2 Table . Analytical details showing the cpDNA regions used in the study, their respective primers sequences, PCR conditions, and product sizes.

| Region / code | Primer sequences (5’→3’) | PCR conditions | Approximate length [bp] | Reference |
| --- | --- | --- | --- | --- |
| *trnQ-rps16* (cpDNA01) | F: GCGTGGCCAAGYGGTAAGGC R: GTTGCTTTYTACCACATCGTT | 95 ºC – 4 min 95 ºC – 20 sec 61 ºC (-0.3 ºC/cycle) – 30 sec 72 ºC – 90 sec (10 ×) 95 ºC – 20 sec 58 ºC – 30 sec 72 ºC – 90 sec (35 ×) 72 ºC – 5 min | 1900 | (Shaw et al., 2007; Call et al., 2015) |
| *ndhF-rpl32* (cpDNA02) | F: GAAAGGTATKATCCAYGMATATT R: CCAATATCCCTTYYTTTYCCAA | 95 ºC – 4 min  95 ºC – 20 sec  55 ºC (-0.3 ºC/cycle) – 30 sec 72 ºC – 90 sec (10 ×)  95 ºC – 20 sec  52 ºC – 30 sec 72 ºC – 90 sec (35 ×)  72 ºC – 5 min | 1300 | (Shaw et al., 2007; Call et al., 2015) |
| *rps16* (cpDNA03) | F: GTGGTAGAAAGCAACGTGCGACTT R: TCGGGATCGAACATCAATTGCAAC | 95 ºC – 4 min  95 ºC – 20 sec  58 ºC (-0.3 ºC/cycle) – 30 sec 72 ºC – 90 sec (10 ×)  95 ºC – 20 sec  55 ºC – 30 sec 72 ºC – 90 sec (35 ×)  72 ºC – 5 min | 1000 | (Oxelman, Lidén, and Berglund, 1997; Call et al., 2015) |
