# Supplementary material for: Haplotyping of Cornus florida and C. kousa chloroplasts: Insights into species-level differences and patterns of plastic DNA variation in cultivars
Source: PLoS One. 2018 Oct 23;13(10):e0205407. doi: 10.1371/journal.pone.0205407 (PMC6198962; doi:10.1371/journal.pone.0205407)
Supplement: S3 Table — Reported % values (in parentheses–base pair length of the alignment) for aligned cpDNA01, 02, 03, and concatenated sequences. Sequenced samples are listed out in Table 1. Sequences were aligned using MAFFT without G regions trimming of uninformative sites. Cornus species grouping as in ref. [2]. (DOCX) [file pone.0205407.s006.docx]

S3 Table. Simple sequence repeats (SSRs) present in the cpDNA of *Cornus controversa* (KU852492.1; NC_030260.1 voucher SCONT20150712 chloroplast, complete genome, 158,674 bp), pose little alternative to chlorotyping. The SSRs were detected using the https://ssr.nwisrl.ars.usda.gov with the default settings.

| SSR Number | Start^A^ | End^A^ | Type^B^ | Motif^B^ | Motif Length^C^ | # Repeats^C^ | Compound Motif^D^ |
| --- | --- | --- | --- | --- | --- | --- | --- |
| 1 | 9546 | 9564 | i,a |  |  | 6 | (CAA)_3_-(AAG)_3_ |
| 2 | 44706 | 44728 | i,a |  |  | 5 | (AAT)_3_-(ATAAATA)_2_ |
| 3 | 49379 | 49403 | i,a |  |  | 16 | (TA)_7_-(TATATATAT)_2_-(TATATAT)_2_-(TA)_5_ |
| 4 | 50975 | 50990 | p | at | 2 | 8 | (AT)_8_ |
| 5 | 53611 | 53625 | p | tta | 3 | 5 | (TTA)_5_ |
| 6 | 59934 | 59951 | i,a |  |  | 5 | (ACTTATT)_2_-(TTA)_3_ |
| 7 | 70319 | 70337 | i,a |  |  | 5 | (CATATAA)_2_-(ATA)_3_ |
| 8 | 71029 | 71044 | i,a |  |  | 5 | (TTATTAT)_2_-(TTA)_3_ |
| 9 | 98163 | 98191 | i,a |  |  | 4 | (GATCATCA)_2_-(AGATTAT)_2_ |
| 10 | 116798 | 116812 | i,a |  |  | 5 | (TAA)_3_-(AATAATA)_2_ |
| 11 | 148333 | 148359 | i,a |  |  | 4 | (TATAATC)_2_-(ATCTGATG)_2_ |

^A^ Numeric position of the beginning/end of the found SSR in the complete cpDNA sequence deposited.

^B^ Type ("i,a" (imperfect, asymmetrical); "p" (perfect)) of the SSR. For perfect SSRs, the motif found.

^C^ For perfect SSRs, the length of the found motif. For imperfect SSRs, this is left blank. For perfect SSRs, the number of repeats of the single motif. For imperfects SSRs, the total number of repeats of all motifs combined.

^D^ The SSR in (motif)count-(motif)count-motif(count)... format.
