# Supplementary material for: Haplotyping of Cornus florida and C. kousa chloroplasts: Insights into species-level differences and patterns of plastic DNA variation in cultivars
Source: PLoS One. 2018 Oct 23;13(10):e0205407. doi: 10.1371/journal.pone.0205407 (PMC6198962; doi:10.1371/journal.pone.0205407)
Supplement: S4 Table — The SSRs were detected in the sequence (KU852492.1; NC_030260.1 voucher SCONT20150712 chloroplast, complete genome, 158,674 bp) using the https://ssr.nwisrl.ars.usda.gov with the default settings. (DOCX) [file pone.0205407.s007.docx]

S4 Table. Proportion of variable sites in the MAFFT-aligned sequences. Reported % values (in parentheses – base pair length of the alignment) for aligned cpDNA01, 02, 03, and concatenated sequences. Sequenced samples are listed out in Table 2. Sequences were aligned using MAFFT without G regions trimming of uninformative sites.

|  | cpDNA01 | cpDNA02 | cpDNA03 | cpDNA010203 |
| --- | --- | --- | --- | --- |
| *C. florida* | 2% (1693 bp) | 11% (1011 bp) | 7% (911 bp) | 6% (3615 bp) |
| *C. kousa* | 12% (1749 bp) | 9% (1011 bp) | 7% (918 bp) | 11% (3715 bp) |
| Big-Bracted | 20% (1825 bp) | 24% (1054 bp) | 14% (920 bp) | 41% (3806 bp) |
| Cornelian Cherries | 7% (1549 bp) | 11% (1152 bp) | 1% (910 bp) | 35% (3611 bp) |
| Blue-/White-Fruited | 30% (1727 bp) | 26% (1079 bp) | 2% (909 bp) | 45% (3788 bp) |
| All | 62% (1975 bp) | 52% (1160 bp) | 19% (941 bp) | 64% (4217 bp) |
